# Polyphyllin I suppresses the gastric cancer growth by promoting cancer cell ferroptosis

**DOI:** 10.3389/fphar.2023.1145407

**Published:** 2023-04-04

**Authors:** Fang Zheng, Yeshu Wang, Qunfang Zhang, Qiuyuan Chen, Chun-Ling Liang, Huazhen Liu, Feifei Qiu, Yuchao Chen, Haiding Huang, Weihui Lu, Zhenhua Dai

**Affiliations:** ^1^ Section of Immunology, The Second Affiliated Hospital of Guangzhou University of Chinese Medicine, Guangzhou, Guangdong, China; ^2^ Joint Immunology Program, Guangdong Provincial Academy of Chinese Medical Sciences, Guangzhou, Guangdong, China

**Keywords:** ferroptosis, gastric cancer, NRF2, polyphyllin I, anticancer drug

## Abstract

**Background:** Ferroptosis is a new form of regulated cell death characterized by the accumulation of iron-dependent lipid peroxides and membrane damages. Recent studies have identified an important role for cancer cell ferroptosis in antitumor therapy. On the other hand, polyphyllin I (PPI) has been reported to exert antitumor effects on some types of cancers. However, it remains unknown whether or not PPI regulates cancer cell ferroptosis.

**Methods:** Two types of human gastric cancer cells (AGS and MKN-45) were used to establish tumor xenograft models in nude mice that were treated with polyphyllin I (PPI) to observe tumor growth, while cells also were cultured for *in vitro* studies. Ferroptosis, based on the intracellular ROS/lipid ROS production and accumulation of ferrous ions, was detected using a fluorescence microscope and flow cytometer, while the expression of NRF2/FTH1 was measured using Western blotting assays.

**Results:** Here we found that PPI inhibited the gastric cancer growth *in vivo* and *in vitro* while increasing the intracellular reactive oxygen species (ROS)/lipid peroxides and ferrous ions in the gastric cancer cells. PPI also decreased the levels of nuclear factor erythroid 2-related factor 2 (NRF2) and ferritin heavy chain 1 (FTH1) in gastric cancer cells *in vitro*. Moreover, liproxstain-1, an inhibitor of cell ferroptosis, mostly reversed the cell ferroptosis and tumor growth arrest induced by PPI. Finally, the effects of PPI on cancer cell ferroptosis were diminished by the overexpression of NRF2.

**Conclusion:** For the first time, our results have demonstrated that PPI exerts its antitumor activity on the gastric cancer by, at least partially, inducing cancer cell ferroptosis *via* regulating NRF2/FTH1 pathway. These findings may be implicated for clinical replacement therapy of the gastric cancer.

## Introduction

As a new form of regulated cell death distinct from conventional apoptosis, ferroptosis is caused by iron-dependent lipid peroxidation and subsequent cell membrane damage (Dixon et al., 2012). Increased iron accumulation, free radical production, and lipid peroxidation are critical for the induction of ferroptosis (Dixon et al., 2012; Tang et al., 2021). Although the links between ferroptosis and carcinogenesis and its roles in anticancer therapy have been extensively studied recently, the molecular mechanisms underlying ferroptosis remain poorly understood. Previous studies have revealed that ferroptosis plays a vital role in tumor occurrence and that abundant cell ferroptosis can inhibit tumor progression (Lei et al., 2022). Ferroptosis-inducing drugs may lead to cancer cell death or tumor growth arrest and affect the efficacy of chemotherapy, radiotherapy, targeted therapy, and even immunotherapy (Chen et al., 2021a; Zhang et al., 2022; Zhao et al., 2022). Therefore, agents targeting ferroptosis pathway could provide new a therapeutic strategy for treating various cancers.

Polyphyllin I (PPI), a natural ingredient extracted from the root of *Paris polyphylla*, has been shown to exert antitumor effects on various cancers, including the gastric cancer (Tian et al., 2020a). Polyphyllin series, including PPI, can be derived from *Rhizoma paridis* and are also anti-tumorous (Li et al., 2023). Mechanistically, PPI activated the AMPK or p21/CDK2/Rb pathway to inhibit the growth of non-small cell lung cancer (Wu et al., 2020; Shen et al., 2021), suppressed the tumoral angiogenesis through the Twist1/VE-cadherin pathway, and induced apoptosis in hepatocellular carcinoma (Shi et al., 2015; Xiao et al., 2018; Zeng et al., 2020). Furthermore, it induced autophagy and apoptosis in the gastric cancer and colon cancer cells by inhibiting the PDK1/Akt/mTOR signaling (He et al., 2019; Luo et al., 2022). The mechanisms underlying antitumor effects of PPI are associated with the inhibition of cell proliferation and invasion, induction of cell cycle arrest and apoptosis, promotion of autophagy, and enhancement of tumor sensitivity to the chemotherapy or other targeted therapy. The transcriptional and signal pathways initiated by PPI may include STAT3, AMPK, FOXO3 and PI3K/Akt, etc. (Han et al., 2015; Li et al., 2016; Lou et al., 2017; Liu et al., 2018; Han et al., 2020; Li et al., 2020; Lai et al., 2021). However, there has been no any report on a potential impact of PPI on cancer cell ferroptosis.

In this study, we investigated the effects of PPI on the gastric cancer using two human cancer cell lines, AGS and MKN-45. Indeed, we found that PPI inhibited the gastric cancer growth *in vitro* and *in vivo*. Importantly, for the first time, we demonstrated that PPI induced gastric cancer cell ferroptosis, as evidenced by the accumulation of ROS/lipid peroxides and enrichment of ferrous ions in the gastric cancer cells treated with PPI. Further studies revealed that NRF2/FTH1 pathways played an important role in PPI-induced cell ferroptosis in the gastric cancer. These findings suggest that PPI may serve as a novel inducer of ferroptosis for a replacement therapy of the gastric cancer.

## Materials and methods

### Animals, tumor models and treatment of mice

BALB/c nude mice (female, 6-8* *weeks old, body weight 18-20* *g) were obtained from Experimental Animal Center of Guangdong Province (Guangzhou, China). The mice were housed in a specific pathogen-free (SPF) animal facility with controlled conditions. All animal experiments complied with the National Institutes of Health guides for the care and use of laboratory animals (NIH Publications No. 8023, revised 1978), while animal protocols were approved by the Institutional Animal Care and Use Committee of the Second Affiliated Hospital of Guangzhou University of Chinese Medicine. At the end of experiments, mice were euthanized using overdoses of Pelltobarbitalum natricum.

A subcutaneous gastric tumor model was established by subcutaneously injecting 1×10^6^ AGS cells or 2×10^6^ MKN-45 cells near the right axilla of mice. Seven days after tumor cell inoculation, mice received daily i. p. Injection of PPI (3 mg/kg, dissolved in 1% DMSO +5% PEG300 + 5% Tween 80 + 89% deionized water), as described previously [26], or the control solution with the same solvent. Mice were weighed at day 15, while tumor volumes were measured every 3 days and calculated using a formula: length X width ^2^/2.

Furthermore, for the experiments with liproxstain-1 that blocks ferroptosis, 1×10^6^ AGS cells were subcutaneously injected into BALB/c nude mice. Mice then were randomly divided into 4 groups (6 mice/group): controls, PPI (3 mg/kg), Liproxstain-1 (30 mg/kg), and both (*i.p*. daily). Similarly, the tumor volumes were measured every 2-3 days.

### Drugs and reagents

Polyphyllin I (PPI) was purchased from Meilun Biotechnology Co., Ltd. (Lot number: MB7074, Dalian, China, purity>98%), while Liproxstatin-1 was obtained from Selleck Chemical (Lot#: S7699, Houston, United States). 3-(4,5-Dimethylthiazol-2-yl)-2,5-diphenyltetrazolium bromide (MTT) powder was a product from Sigma Aldrich (St. Louis, United States). Monoclonal antibodies specific for NRF2 and FTH1 were purchased from Cell Signaling Technology Inc. (Danvers, United States) and Abcam (China Branch, Shanghai, China), respectively.

### Cell lines and cultures

Human gastric cancer cell line, MKN-45, was provided by Chinese Academy of Sciences Cell Bank of Type Culture Collection (Shanghai, China), while AGS human cell line was purchased from American Type Culture Collection (ATCC, Manassas, United States). These cells were cultured in RPMI-1640 medium (Gibco, United States), supplemented with 10% (v/v) fetal bovine serum (Hyclone, United States), 100 μg/mL streptomycin and 100* *U/mL penicillin (Gibco), in a humidified incubator containing 5% CO2 at 37°C. Cells with passage numbers of 15–20 were used for all experiments.

### MTT assays evaluating cell growth or expansion *in vitro*

Cell growth or expansion was tested using the 3-(4, 5-dimethylthiazol-2-yl)-2, 5- diphenyltetrazolium bromide (MTT) dye reduction methods. After the culture with PPI, the cells were incubated with the medium containing 10% MTT solution (5 mg/mL in PBS) at 37°C for 4 h. The absorption was measured at 570 nm using a microplate reader (Perkin Elmer, Victor X5, United States). The cell viability (%) was calculated based on a formula: (absorbance of test sample/absorbance of control) ×100%.

### Determination of intracellular levels of ROS and lipid peroxidation

To measure ROS production, we labeled AGS and MKN-45 cells with a DCFH-DA fluorescent probe (MCE, United States). Briefly, cells were washed after treatment with PPI and stained with 1 mL of DCFH-DA (10 μM, in HBSS) at 37°C for 30 min. Cells were then washed and re-suspended in HBSS. The fluorescence intensity (MFI) of DCFH-DA was measured using a flow cytometer (ACEA, Novo Quanteon, United States).

To measure lipid-ROS levels, a C11-BODIPY fluorescent probe (ThermoFisher, United States) was used to determine lipid peroxidation. After the treatment, cells were incubated in HBSS containing 5 μM C11-BODIPY at 37°C for 30 min and harvested for flow cytometric analyses. Mean fluorescence intensity (MFI) was used to represent lipid-ROS levels.

### Detection of intracellular Fe2+ ions

To measure the levels of intracellular Fe2+ ions, we used fluorescent probes to label Fe2+ in cells. Briefly, AGS and MKN-45 cells were seeded in 48-well and 6-well plates, respectively, at a density of 1 × 10^5^ cells/mL and incubated overnight. After different treatments, cells were then washed and incubated in HBSS solution containing FerroOrange (Dojindo, Japan) with a concentration of 1 μM at 37°C in a 5% CO2 incubator for 30 min. Cells were then observed under an inverted fluorescence microscope, with fluorescence images acquired immediately. Additionally, cells were harvested and resuspended in HBSS for flow cytometric analyses to further quantify intracellular Fe2+ ion levels.

### Western blotting

We determined the expression of FTH1 and NRF2 proteins *via* Western blot analyses. Briefly, protein lysates were prepared from cultured cells or mouse tumor tissue using an ice-cold cell lysis buffer (ThermoFisher, United States) supplemented with a protease inhibitor cocktail (Roche, Switzerland). Equal amounts of proteins were separated by 12% SDS polyacrylamide gels using a vertical electrophoresis system (Bio-Rad, United States), and then proteins were transferred to PVDF membranes (Millipore, United States) using semi-dry transfer (Bio-Rad). After blocking with 5% BSA, the membranes were incubated with primary antibodies, including FTH1 (CST, 1:1000), NRF2 (Abcam, 1:1000) or GAPDH (Abcam, 1:10000), at 4°C overnight, followed by incubation with secondary antibodies for 1 hour at room temperature. Protein levels were detected using ChemiDoc XRS + Imagine System (Bio-Rad, United States), while data were analyzed using the Image Lab software.

### Statistical analysis

Statistical analyses were carried out using GraphPad Prism 8.0 software (La Jolla, CA, United States). All data were analyzed using the unpaired *t*-test or One-way ANOVA. Asterisks shown in the figures indicate significant differences in comparisons of experimental groups with the corresponding controls. The statistical significance was assumed at a value of *p* < 0.05.

## Results

### PPI inhibits the gastric cancer growth

**FIGURE 1:**
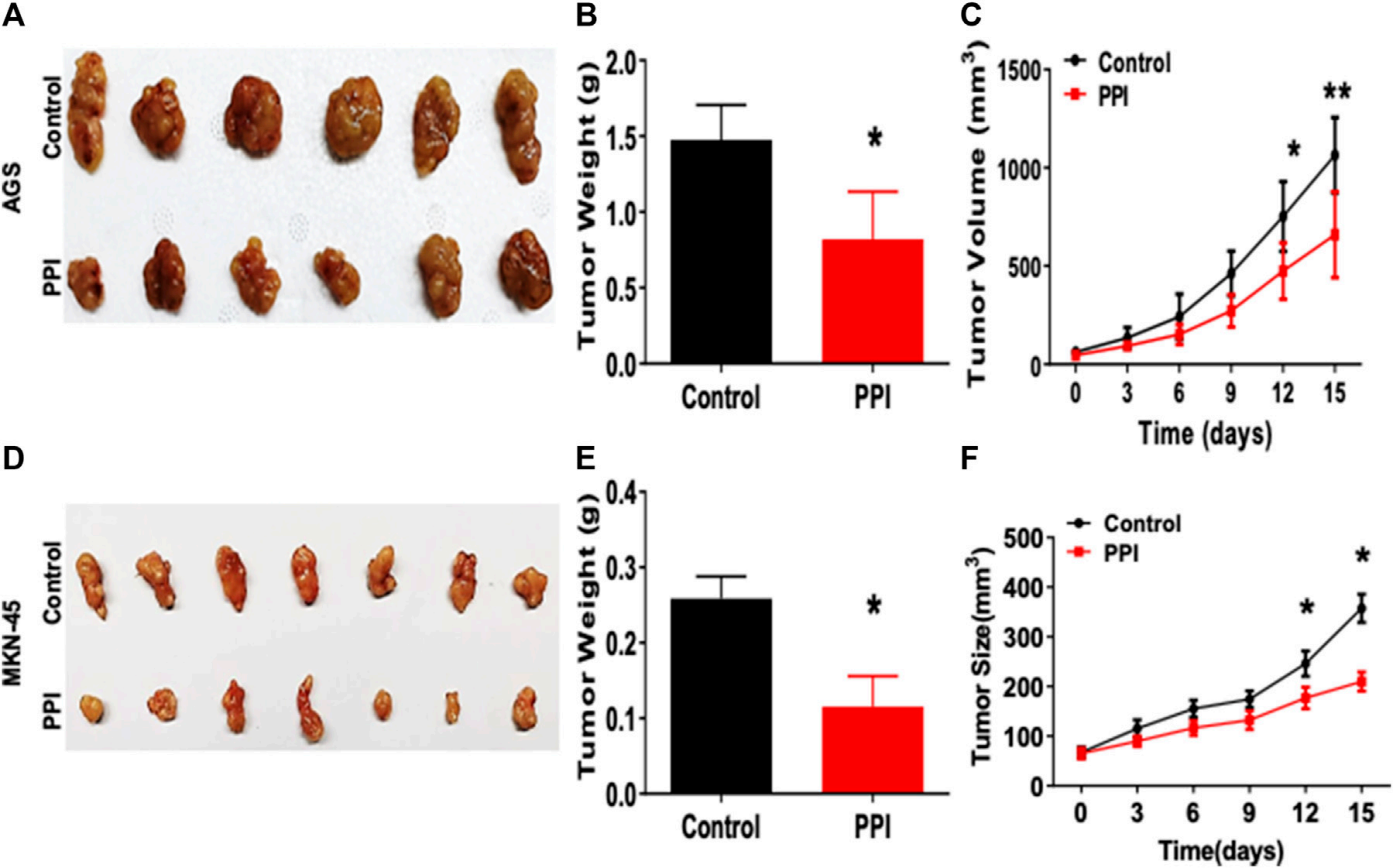
PPI inhibits the tumor growth of AGS and MKN-45 cells in BALB/c nude mice. BALB/c nude mice were injected subcutaneously with 1 × 10^6^ AGS or 2 × 10^6^ MKN-45 human tumor cells. One week after inoculation, mice were administrated with the solvent (Control) or PPI (3 mg/kg/day) for up to 15 days. **(A)** Tumor xenografts (AGS) were harvested at day 15. Shown are photos of the tumors derived from control vehicle- and PPI-treated mice. **(B)** Then, the tumor weights were also measured at day 15. **(C)** The tumor volumes were determined at various time points. **(D–F)** Similarly, Tumors (MKN-45) also were harvested for taking tumor photos **(D)** and measuring tumor weights **(E)** as well as tumor volumes **(F)**. Data are presented as mean ± SD (*n* = 6 mice/group) and analyzed using the unpaired-*t* test (*p < 0.05).

Here, we tested the efficacy of PPI on the gastric cancer using two xenograft tumor models. AGS and MKN-45 cancer cells were subcutaneously injected into the nude mice. Tumor-bearing mice were treated with PPI. We then assessed the tumor volumes and weights of the mice (Figure 1). We found that the AGS tumor weight of PPI-treated mice was significantly reduced compared to that of the control group 15 days after inoculation (Figures 1A, B). Mice treated with PPI also exhibited smaller AGS tumor volumes than did the control group on days 12 and 15, respectively (Figure 1C). Similar findings were observed in MKN-45 tumors (Figures 1D–F). These findings confirmed that PPI indeed inhibits the gastric cancer growth.

### PPI inhibits the growth of gastric cancer cells *in vitro* and induces their ferroptosis

**FIGURE 2:**
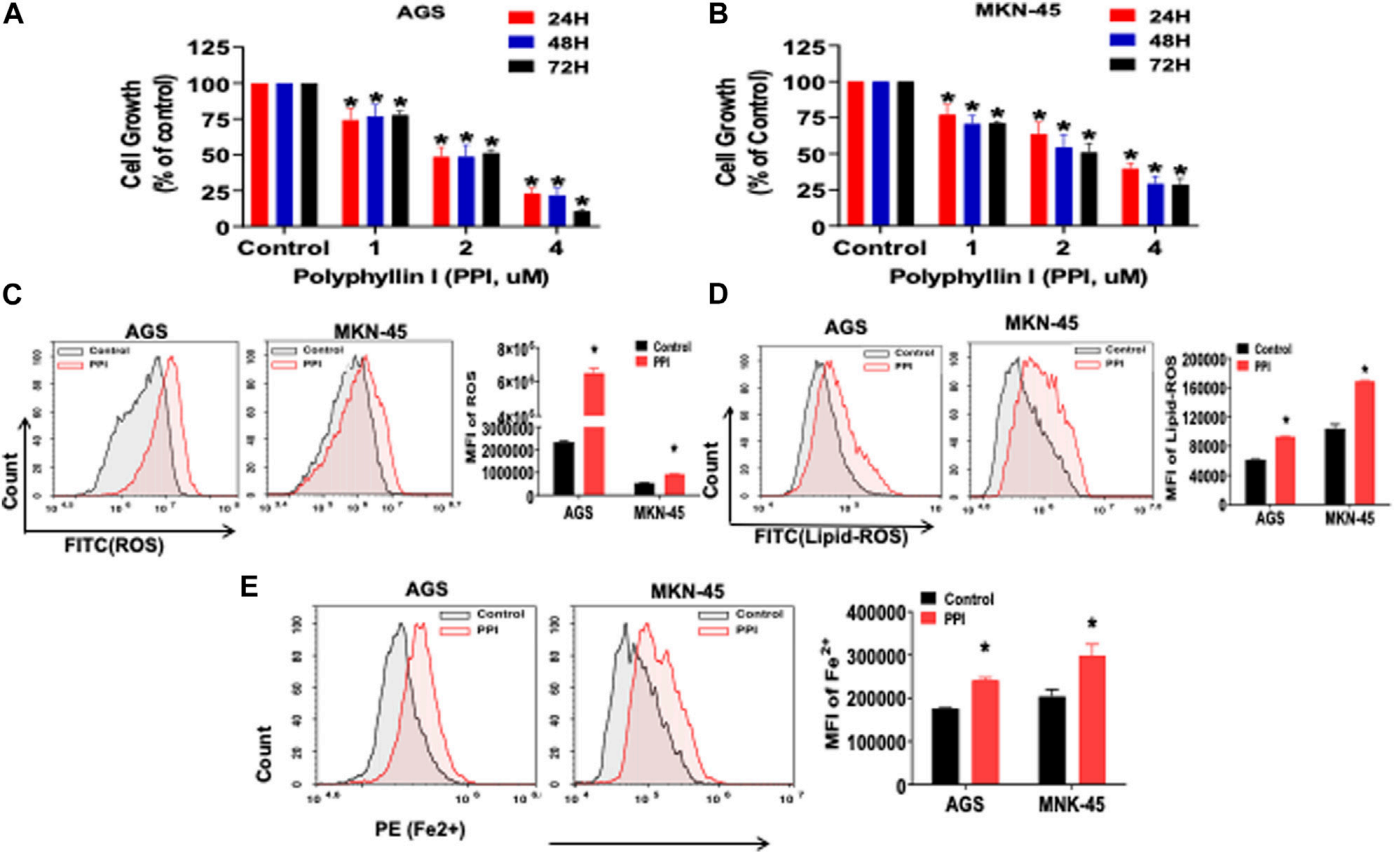
PPI suppresses cell growth and induces ferroptosis in the gastric cancer cells. **(A, B)** The cell growth (expansion) of AGS and MKN-45 cells after treatment with PPI (0, 1, 2, 3, 4 μM, for 24, 48 and 72 h, respectively) was examined using MTT assays. **(C, D)** The levels of cellular ROS and lipid peroxides (lipid-ROS) after the treatment with PPI (3 μM) for 24 h in AGS and MKN-45 cells were analyzed using a flow cytometer. **(E)** The levels of intracellular ferrous ions (Fe^2+^) in AGS and MKN-45 cells after PPI treatment for 24 h were quantified using a flow cytometer. Data are shown as mean ± SD while *p* values were determined using one-way ANOVA (*p < 0.05).

Previous reports showed that PPI inhibited the tumor growth *via* several mechanisms (Dong et al., 2018; Zhang et al., 2018; Han et al., 2020). In this study, we first confirmed that PPI indeed suppressed both AGS and MKN-45 cancer cell growth *in vitro* in a dose-dependent manner based on MTT assays (Figure 2A, B). We then determined the effects of PPI on the levels of intracellular reactive oxygen species (ROS), lipid peroxidation and ferrous ions in the gastric cancer cells 24 h after cell culture. Our data showed that the intracellular levels of both ROS and Lipid-ROS in the gastric cancer cells treated with PPI were significantly increased compared to the control group (Figures 2C, D). In addition, using a flow cytometer, we revealed that PPI increased the levels of ferrous ions in both AGS and MKN-45 cells (Figure 2E). These findings suggest that PPI promotes the gastric cancer cell ferroptosis.

### A ferroptosis inhibitor largely diminishes the effects of PPI on gastric cancer cell growth and ferroptosis *in vitro*

**FIGURE 3:**
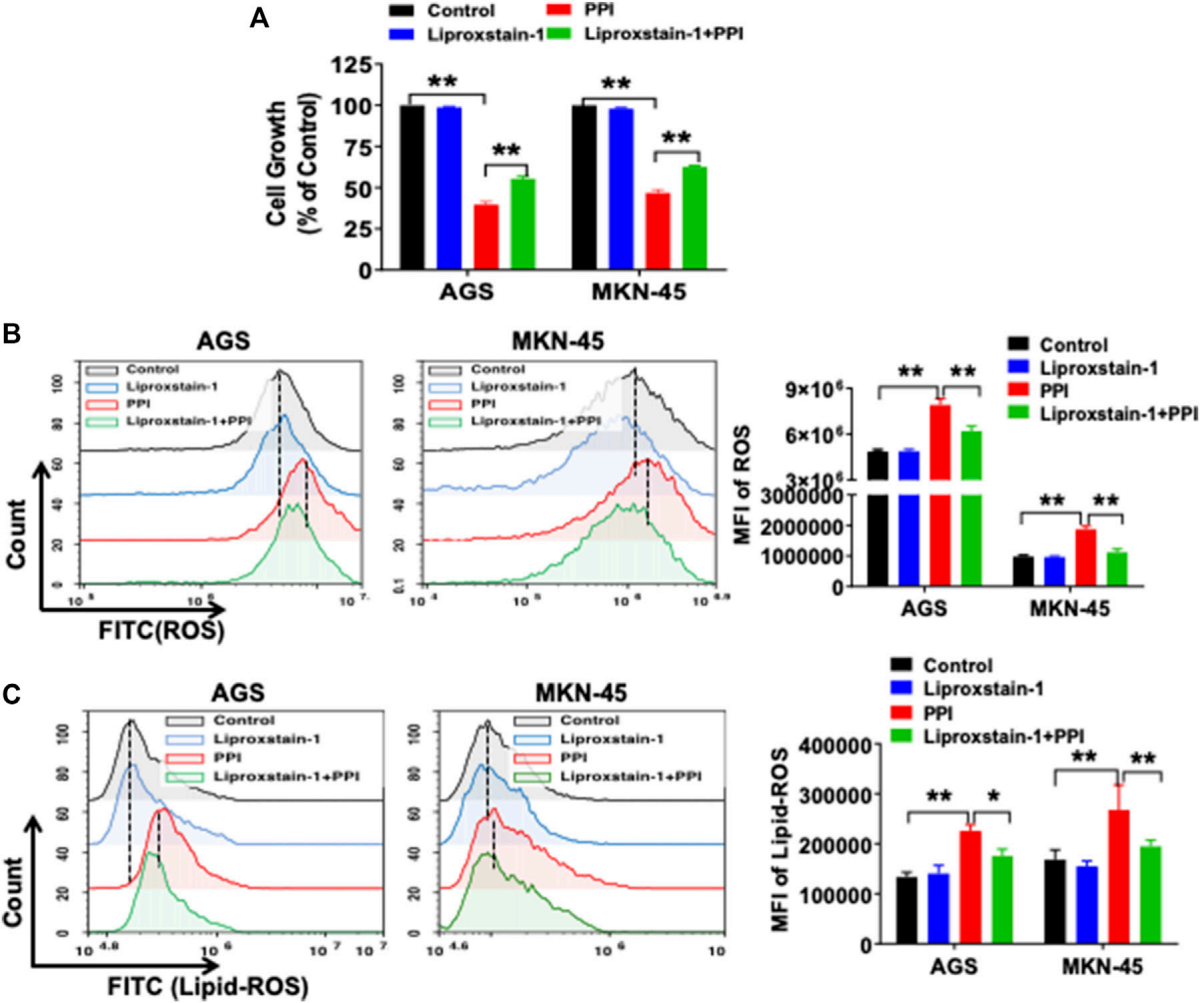
A ferroptosis inhibitor reverses PPI-induced ferroptosis and suppression of gastric cancer cell growth *in vitro*. **(A)** AGS and MKN-45 cells were pretreated with an inhibitor of ferroptosis, liproxstain-1 (200 nM), for 2 h, and then PPI was added for an additional 24 h. Cell growth/expansion was measured *via* MTT assays. **(B and C)** Intracellular ROS and lipid peroxides (lipid-ROS) levels were measured using flow cytometry. Data are shown as mean ± SD. *p* values were determined using one-way ANOVA (*p < 0.05, **p < 0.01).

**FIGURE 4:**
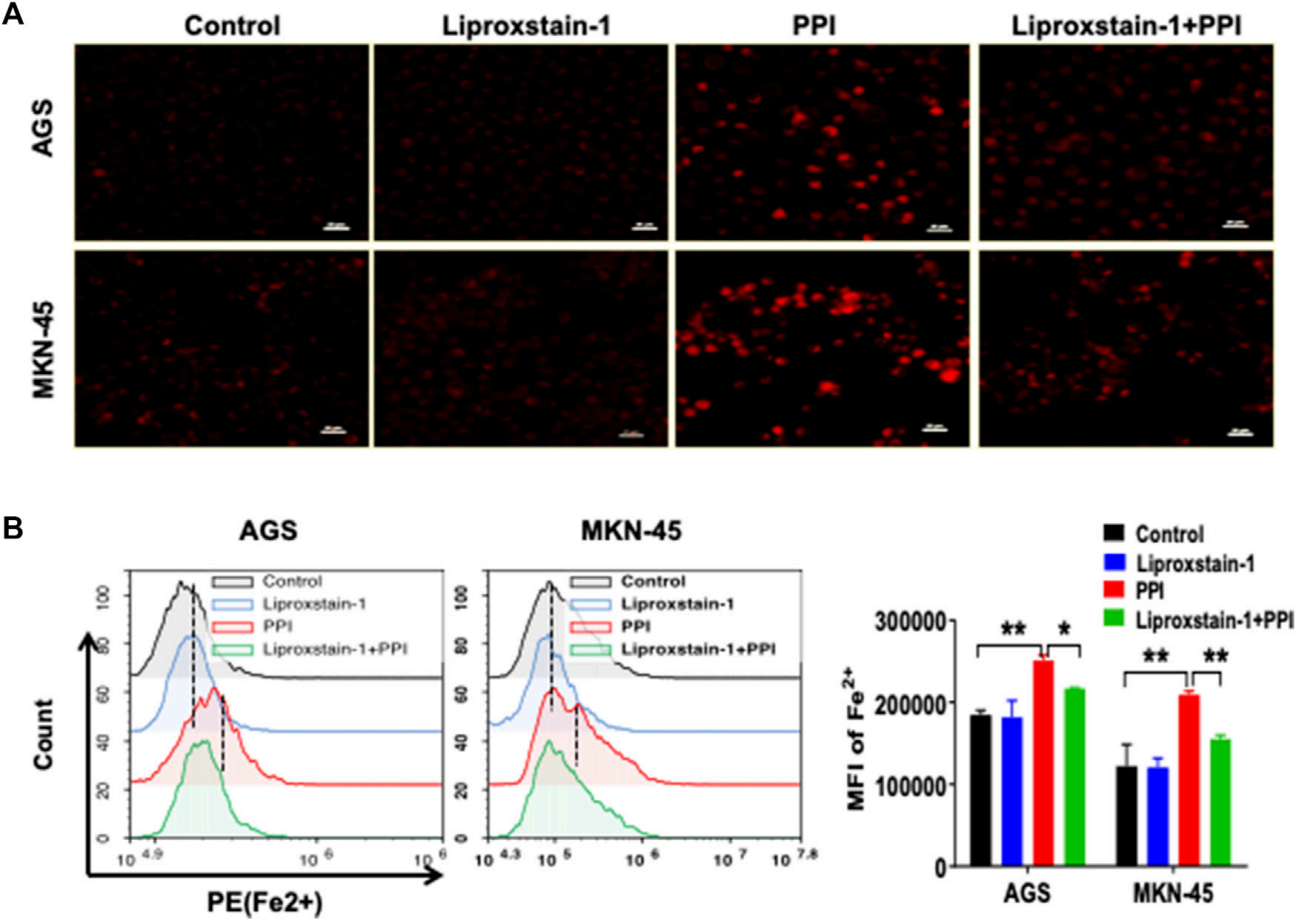
A ferroptosis inhibitor is also confirmed to block PPI-induced accumulation of Fe2+ in gastric cancer cells. AGS and MKN-45 cells were treated with liproxstain-1 and/or PPI as described in Figure 3. They were then labeled with FerroOrange fluorescent probes to detect intracellular ferrous (Fe2+) concentrations through fluorescence microscopy **(A)** and flow cytometry **(B)**. Scale bars = 50 µm. Data are shown as mean ± SD while *p* values were determined using one-way ANOVA (*p < 0.05, **p < 0.01).

Ferroptosis is mainly characterized by the accumulation of iron-dependent lipid peroxides, which can be reversed by ferroptosis inhibitors (Li et al., 2021). A specific ferroptosis inhibitor, Liproxstain-1, was used to confirm whether the anticancer effects of PPI were mediated partially by its induction of cell ferroptosis. We found that Liproxstain-1 largely abolished the effects of PPI on cell growth in both AGS and MKN-45 cells (Figure 3A). Furthermore, PPI increased the intracellular ROS and Lipid-ROS levels in the cancer cells whereas these effects of PPI were largely blocked by Liproxstain-1 (Figure 3B, C). In addition, Liproxstain-1 also reduced the PPI-induced accumulation of ferrous ions based on fluorescence staining (Figure 4A) and flow analysis (Figure 4B). These results indicate that PPI inhibits the gastric cancer cell growth by, at least partially, inducing cell ferroptosis.

### Inhibition of ferroptosis by Liproxstain-1 also partially reverses the inhibitory effects of PPI on tumor growth *in vivo*

**FIGURE 5:**
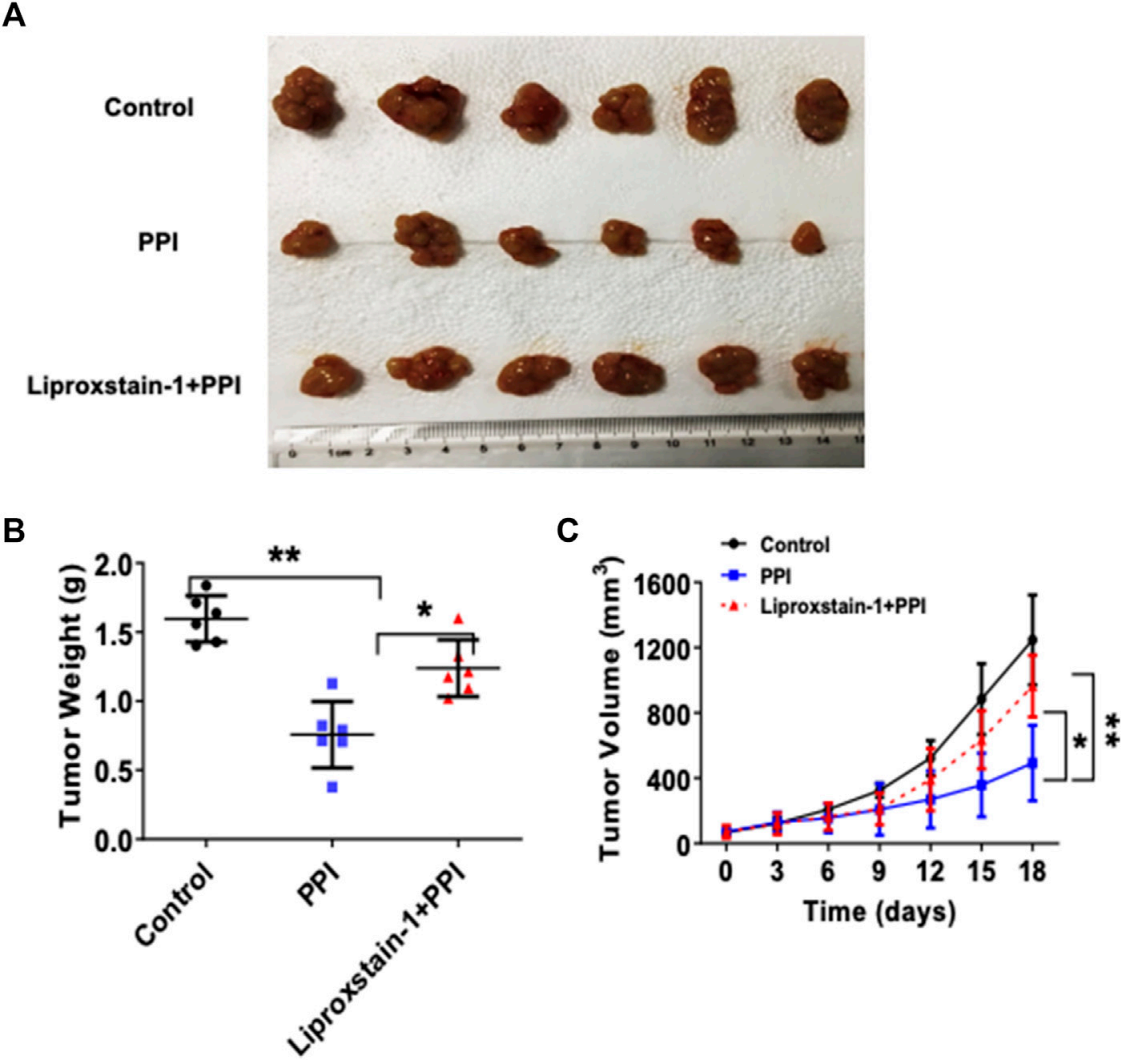
Liproxstain-1 reverses the inhibitory effects of PPI on tumor growth *in vivo*. BALB/c nude mice were subcutaneously injected with 1×10^6^ AGS cells. One week later, the mice were administered with PPI (3 mg/kg/day) alone or in combination with liproxstain-1. **(A)** The tumor xenografts were harvested at day 15, and photographs of tumor xenografts were taken (*n* = 6 mice/group). **(B)** The weights of tumors derived from the nude mice were also measured at day 15. **(C)** Finally, tumor volumes were determined at various time points for up to 18 days. Data are presented as mean ± SD (*n* = 6 mice/group, *p < 0.05; and **p < 0.01).

To further determine if the effects of PPI on the gastric cancer growth *in vivo* are also dependent on its induction of ferroptosis, we administrated Liproxstain-1 and/or PPI in tumor-bearing mice. As expected, PPI indeed reduced the tumor weights (Figure 5A, B). However, we found that Liproxstain-1 treatment reversed, at least in part, the tumor weight loss induced by PPI treatment (Figure 5A, B). Similarly, treatment with Liproxstain-1 also blocked the inhibitory effects of PPI on the tumor growth based on the tumor volumes at various time points (Figure 5C). These results further suggest that PPI inhibits the gastric cancer growth *in vivo* by, at least partially, inducing cancer cell ferroptosis.

### PPI downregulates NRF2 and FTH1 in the gastric cancer cells

**FIGURE 6:**
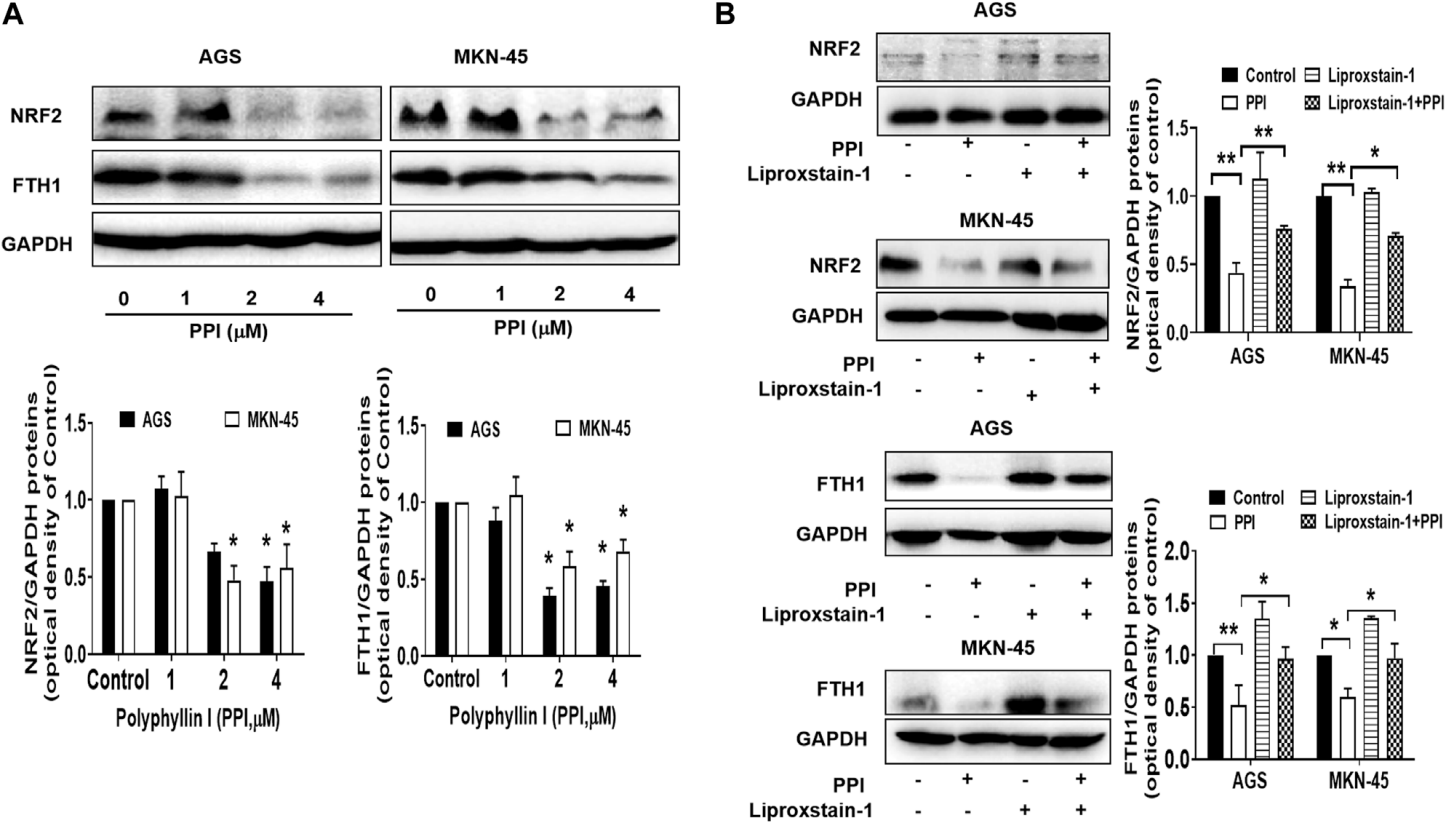
Effects of PPI on the expression of NRF2 and FTH1 in gastric cancer cells. AGS and MKN-45 cells were cultured and treated with PPI at various concentrations for 24 h, and then the protein levels of NRF2 and FTH1, without **(A)** or with **(B)** Liproxstain-1 treatment, were detected using Western blot assays. Data are presented as mean ± SD, while *p* values were determined using one-way ANOVA (*p < 0.05, and **p < 0.01, as indicated).

Nuclear factor erythroid 2-related factor 2 (NRF2) is a specific transcription factor that plays a role in mitigating lipid peroxidation and ferroptosis (Dodson et al., 2019). On the other hand, ferritin heavy chain 1 (FTH1), a heavy subunit of ferritin, is also a key regulator of ferroptosis (Tian et al., 2020a; Fang et al., 2021). Using Western blot assays to detect ferroptosis-related proteins in the cancer cells after PPI treatment *in vitro*, we found that PPI downregulated the expression of both NRF2 and FTH1 (Figure 6A) in both AGS and MKN-45 cancer cells, whereas Liproxstain-1 largely reversed this effect of PPI (Figure 6B), indicating that PPI-induced ferroptosis in the gastric cancer cells is associated with its regulation of NRF2/FTH1 pathway.

### PPI induces ferroptosis of the gastric cancer cells by downregulating NRF2

**FIGURE 7:**
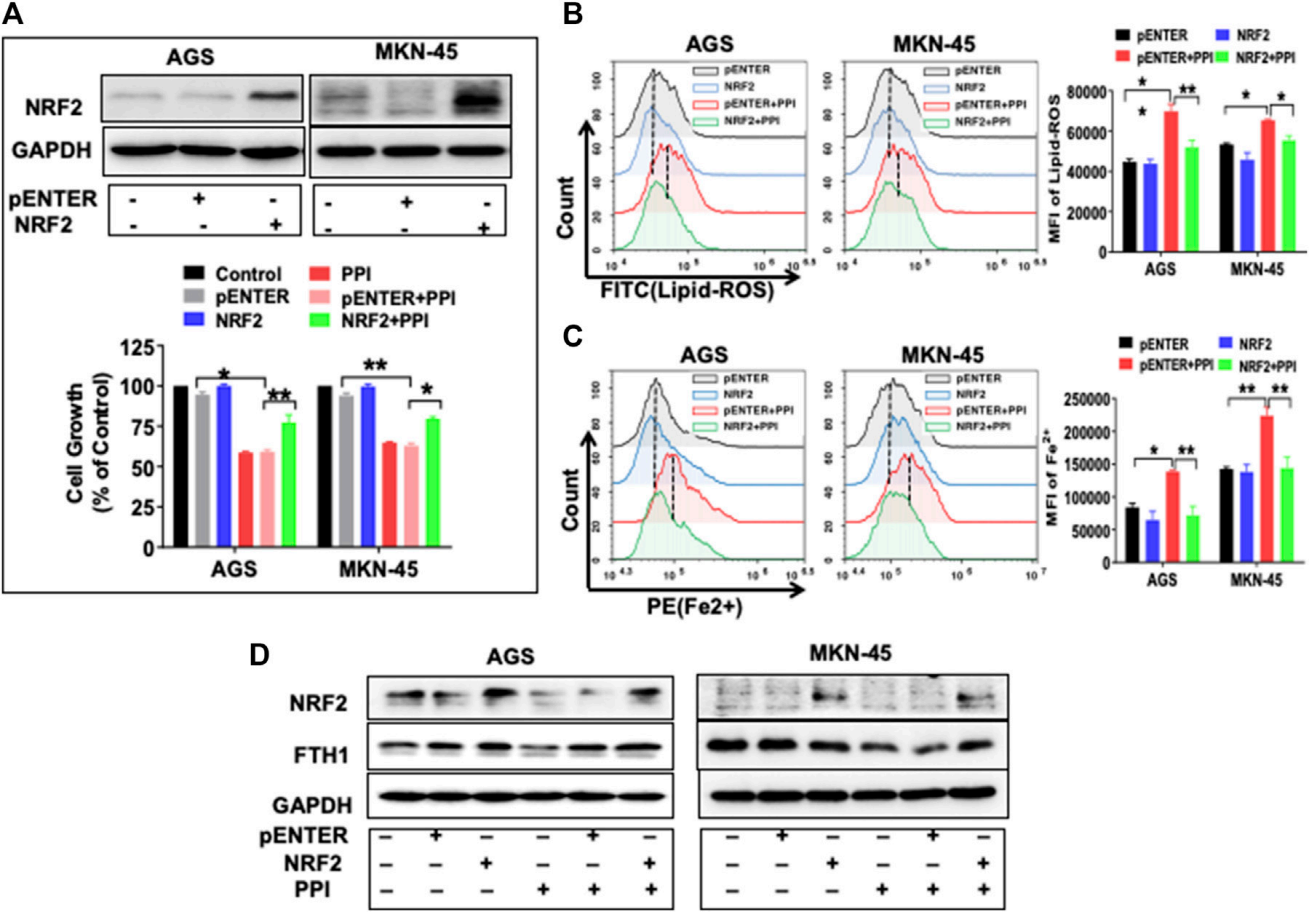
NRF2 overexpression reverses the effects of PPI on cell growth and ferroptosis in the gastric cancer cells. AGS and MKN-45 cells were transfected with NRF2 plasmid or the pENTER control vector for 24 h before they were exposed to PPI (3 μM) for an additional 24 h **(A)** NRF2 expression was confirmed after the transfection, while cell growth was assessed using MTT assays. **(B and C)** The levels of intracellular lipid peroxides (lipid-ROS) and ferrous ions (Fe^2+^) were detected using flow cytometry after the transfections and/or PPI treatment. Data are shown as mean ± SD while *p* values were determined through one-way ANOVA (*p < 0.05, **p < 0.01, compared with pENTER-vector control). **(D)** The expression of NRF2/FTH1 was analyzed using Western blot. Shown is one set of two or three separate experiments.

Previous studies have found that NRF2 controls key signaling components of ferroptosis at a transcriptional level (Dong et al., 2021; Fu et al., 2021; Guan et al., 2022). We also found that PPI inhibited NRF2 expression in the gastric cancer cells (Figure 6AB). Here we further demonstrated that overexpression of NRF2 partially reversed the inhibitory effects of PPI on gastric cancer cell growth (Figure 7A) and mostly blocked the production of lipid-ROS and accumulation of Fe^2+^ induced by PPI (Figure 7B, C). On the other hand, exogenously increased expression of NRF2 also upregulated FTH1 expression, whereas PPI treatment failed to reduce FTH1 expression in the face of NRF2 overexpression (Figure 7D). Taken together, our findings indicate that NRF2 controls ferroptosis through FTH1, while PPI induces cell ferroptosis and tumor repression *via* suppressing NRF2/FTH1 pathway.

## Discussion

There are currently few effective treatments available for the gastric cancer, especially advanced one. Chemotherapy is still one of the standard treatments for the gastric cancer. Unfortunately, chemotherapy resistance remains to be a major obstacle to the cancer treatment (Santero et al., 2022). More recently, targeted therapy and immunotherapy have provided new promising approaches to the treatment of the gastric cancer. However, their clinical application has been limited due to the relevant side effects, resulting in unsatisfactory therapeutic outcomes (Attia and Smyth, 2021; Basile et al., 2021). Therefore, it’s compelling to seek new therapeutic drugs for efficiently treating the gastric cancer with few side effects. In current study, we found that PPI suppressed the gastric cancer growth *in vivo* and *in vitro*. PPI also induced the gastric cancer cell ferroptosis, whereas suppression of ferroptosis mostly reversed the effects of PPI on the cancer growth. Finally, induction of cancer cell ferroptosis by PPI was largely dependent on its regulation of the NRF2/FTH1 pathway.

Ferroptosis, a novel form of the regulated cell death, is characterized by iron-dependent lipid peroxidation induced by excessive ROS and may be important for overcoming the resistance of tumor cells to chemotherapy (Pu et al., 2022). The accumulation of Fe2^+^ with lipid ROS is one of the hallmarks of ferroptosis and is critical for cell fate (Chen et al., 2021b). On the other hand, P53, a tumor suppressor, can regulate the ferroptosis of tumor cells as well as their autophagy and apoptosis (Ji et al., 2022). It may either negatively or positively regulate cancer cell ferroptosis, depending on its specific roles in reprogramming a metabolic network. Recent studies have also shown that ferroptosis plays an important role in cancer therapy since induction of ferroptosis has emerged as a novel strategy for anticancer treatment (Hassannia et al., 2019; Mou et al., 2019; Li and Li, 2020). Therefore, some studies have been looking for new anticancer drugs simply based on their capacity of inducing cancer cell ferroptosis. Others have developed a novel cancer therapy by combining iron oxide nanoparticles with cancer-selective knockdown of seven key ferroptosis-resistant genes, which exhibited antitumor effects on a variety of cancer cells (Luo et al., 2022). However, agents based on metal irons, especially nano-system, have been in the early stages of development. More extensive studies and even clinical trials are needed to confirm their efficacy (Hu et al., 2022).

As an important source for the development of new anticancer compounds, many natural ingredients extracted from traditional herbs or plants have been shown to enhance ferroptosis in cancer cells (Kong et al., 2021; Wen et al., 2021; Zhang et al., 2022; Shao et al., 2022). Polyphyllin I (PPI), a natural product isolated from the roots of Paris polyphylla, exerts its anticancer effects on different types of cancers (Tian et al., 2020b). However, it remains unknown whether and how PPI induces cancer cell ferroptosis, a new anticancer target. In current study, we found that PPI inhibited the tumor growth of both AGS and MKN-45 cells while increasing their intracellular levels of lipid peroxides and ferrous ions. Subsequently, a ferroptosis inhibitor, liproxstain-1, partially reversed the inhibitory effects of PPI on the gastric cancer cell growth *in vitro* and *in vivo*. Therefore, our findings suggest that PPI can suppress the gastric cancer growth by, at least in part, inducing gastric cancer cell ferroptosis.

FTH1, a heavy subunit of the ferritin, is a key regulator of cell ferroptosis, because its levels regulate susceptibility to ferroptosis (Tian et al., 2020b; Fang et al., 2021). On the other hand, NRF2 and its upstream/downstream signaling pathways also play an important role in the regulation of cell ferroptosis (Dodson et al., 2019). However, the exact regulatory mechanisms underlying their roles in ferroptosis remain unclear. In this study, we found that PPI promoted the gastric cancer cell ferroptosis by regulating the NRF2/FTH1 axis given that the overexpression of NRF2 largely reversed the gastric cancer cell ferroptosis induced by PPI. It remains unknown how PPI alters NRF2/FTH1 axis. It could do so by regulating cell death-associated microRNAs or simply interacting with the antioxidant pathway.

In conclusion, we have demonstrated that PPI inhibits the gastric cancer growth by inducing the cancer cell ferroptosis *via* regulating the NRF2/FTH1 pathway. Our data have provided novel insights into the molecular mechanisms underlying the effects of PPI on the gastric cancer cell growth and ferroptosis. Understanding of mechanisms responsible for cancer cell ferroptosis may help design new drugs for targeted cancer therapy.

## Data Availability

The original contributions presented in the study are included in the article/supplementary material, further inquiries can be directed to the corresponding authors.

## Abbreviations

FTH1, Ferritin heavy chain 1; NRF2, Nuclear factor erythroid 2-related factor 2; PPI, Polyphyllin I; ROS, Reactive oxygen species.
