# Corrigendum: Polyphyllin I suppresses the gastric cancer growth by promoting cancer cell ferroptosis

**DOI:** 10.3389/fphar.2023.1201715

**Published:** 2023-05-03

**Authors:** Fang Zheng, Yeshu Wang, Qunfang Zhang, Qiuyuan Chen, Chun-ling Liang, Huazhen Liu, Feifei Qiu, Yuchao Chen, Haiding Huang, Weihui Lu, Zhenhua Dai

**Affiliations:** ^1^ Section of Immunology, The Second Affiliated Hospital of Guangzhou University of Chinese Medicine, Guangzhou, Guangdong, China; ^2^ Joint Immunology Program, Guangdong Provincial Academy of Chinese Medical Sciences, Guangzhou, Guangdong, China

**Keywords:** ferroptosis, gastric cancer, NRF2, polyphyllin I, anticancer drug

There was an error in Figure 2 and Figure 6 as published. Figure 6A,C were disorganized because the GAPDH control for the same sample (PPI-treated MKN-45 cells) were unnecessarily shown twice. In addition, Figure 2E was already included in **Figure 4A** since both figures were derived from the same experiment. The original Figure 2E has been removed and accordingly replaced with the original Figure 2F, while Figures 6A,C have been combined into a single Figure 6A panel.

**FIGURE 2:**
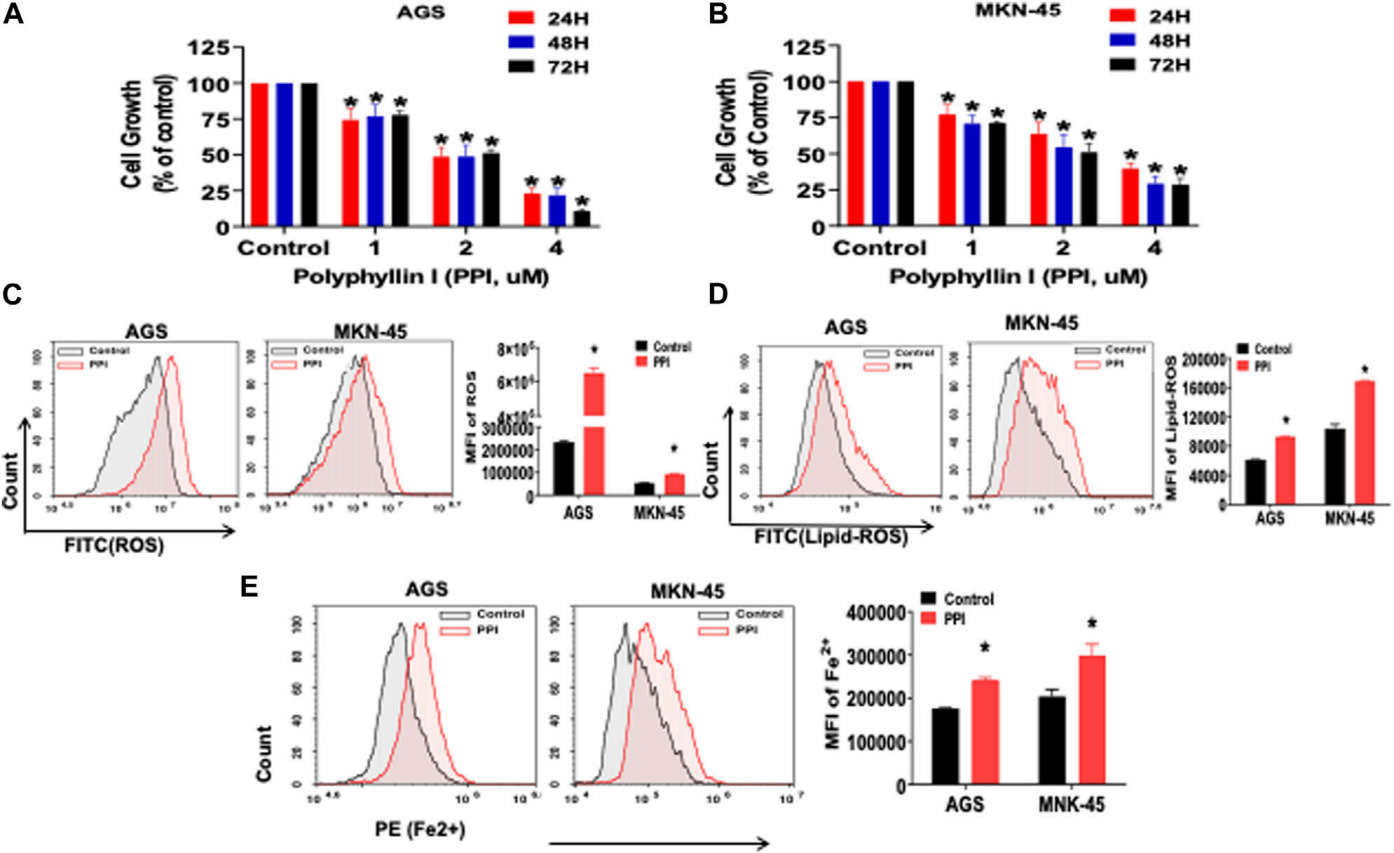
PPI suppresses cell growth and induces ferroptosis in the gastric cancer cells. **(A, B)** The cell growth (expansion) of AGS and MKN-45 cells after treatment with PPI (0, 1, 2, 3, 4 μM, for 24, 48 and 72 h, respectively) was examined using MTT assays. **(C, D)** The levels of cellular ROS and lipid peroxides (lipid-ROS) after the treatment with PPI (3 µM) for 24 h in AGS and MKN-45 cells were analyzed using a flow cytometer. **(E)** The levels of intracellular ferrous ions (Fe^2+^) in AGS and MKN-45 cells after PPI treatment for 24 h were quantified using a flow cytometer. Data are shown as mean ± SD while *p* values were determined using one-way ANOVA (*p < 0.05).

**FIGURE 6:**
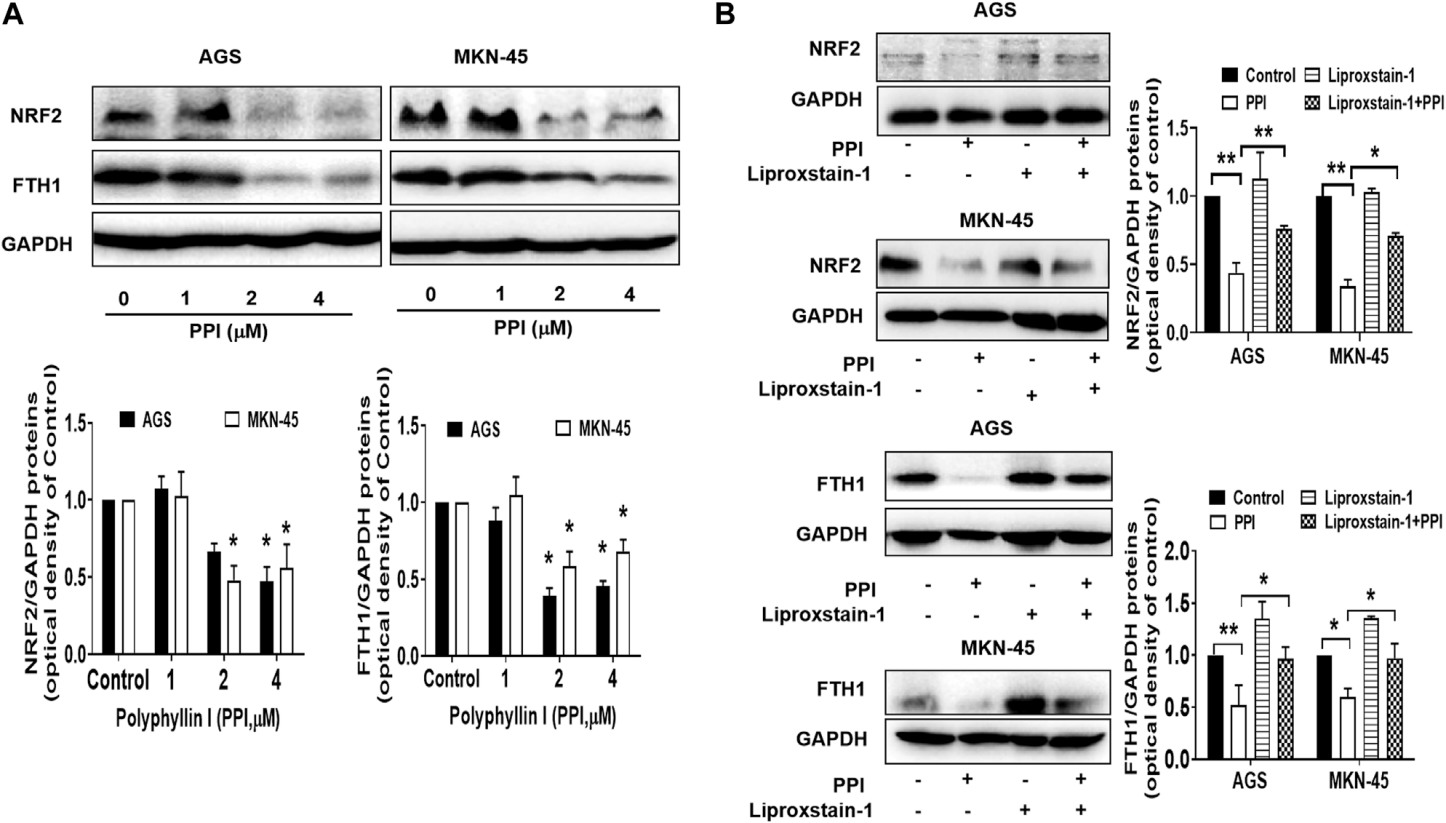
Effects of PPI on the expression of NRF2 and FTH1 in gastric cancer cells. AGS and MKN-45 cells were cultured and treated with PPI at various concentrations for 24 h, and then the protein levels of NRF2 and FTH1, without **(A)** or with **(B)** Liproxstain-1 treatment, were detected using Western blot assays. Data are presented as mean ± SD, while *p* values were determined using one-way ANOVA (*p < 0.05, and **p < 0.01, as indicated).

A correction has been made to the **Results** section, subsection *PPI inhibits the growth of gastric cancer cells in vitro and induces their ferroptosis*: “In addition, using a specific fluorescent probe, FerroOrange, we revealed that PPI increased the levels of ferrous ions in both AGS and MKN-45 cells (Figure 2E,F)” is corrected to “In addition, using a flow cytometer, we revealed that PPI increased the levels of ferrous ions in both AGS and MKN-45 cells (Figure 2E).”

A correction has been made to the **Results** section, subsection *PPI downregulates NRF2 and FTH1 in the gastric cancer cells*: “Using Western blot assays to detect ferroptosis-related proteins in the cancer cells after PPI treatment *in vitro*, we found that PPI downregulated the expression of both NRF2 (Figures 6A,B) and FTH1 (Figures 6C,D) in both AGS and MKN-45 cancer cells, indicating that PPI-induced ferroptosis in the gastric cancer cells is associated with its regulation of NRF2/FTH1 pathway.” is corrected to “Using Western blot assays to detect ferroptosis-related proteins in the cancer cells after PPI treatment *in vitro*, we found that PPI downregulated the expression of both NRF2 and FTH1 (Figure 6A) in both AGS and MKN-45 cancer cells, whereas Liproxstain-1 largely reversed this effect of PPI (Figure 6B), indicating that PPI-induced ferroptosis in the gastric cancer cells is associated with its regulation of NRF2/FTH1 pathway.”.

The corrected Figure 2 and Figure 6, and their captions appear below. The authors apologize for the errors and state that this does not change the scientific conclusions of the article in any way. The original article has been updated.

